# Particulate Matter Pollution in an Agricultural Setting: A Community-Engaged Research Study

**DOI:** 10.3390/environments12100348

**Published:** 2025-09-26

**Authors:** Bonnie N. Young, Jessica Tryner, Luis Hernandez Ramirez, Sherry WeMott, Grant Erlandson, Xiaoying Li, Grace Kuiper, Daniel Alan Dean, Nayamin Martinez, Mollie Phillips, John Volckens, Sheryl Magzamen

**Affiliations:** 1 Department of Environmental and Radiological Health Sciences, Colorado State University, Fort Collins, CO 80523, USA; 2 Access Sensor Technologies, Fort Collins, CO 80524, USA; 3 Department of Mechanical Engineering, Colorado State University, Fort Collins, CO 80523, USA; 4 Central California Environmental Justice Network, Fresno, CA 93727, USA

**Keywords:** air pollution, air quality, community-engaged research, exposure assessment

## Abstract

California’s San Joaquin Valley experiences some of the worst particulate matter (PM) air pollution in the U.S., but PM_2.5_ and PM_10_ exposures in agricultural communities are understudied. We collaborated with rural residents living adjacent to large-scale agricultural production and processing activities to assess 24-h-average personal and indoor PM_2.5_ and PM_10_ concentrations during different seasons. We visited 35 participants from 18 households during December 2023, May 2024, and the September 2024 harvest season to collect PM samples and survey data. Mixed effects linear regression models (with random effects for participant or household) assessed associations between natural log-transformed PM concentrations and regional ambient PM, harvest season, as well as participant/household characteristics. Participants were mostly female (69%) and Hispanic/Latino(a) (100%). Median household distance to processing facility silos was 633 m. Median personal exposures to PM_2.5_ and PM_10_ were 11.1 and 45.5 μg m^−3^. Median indoor PM_2.5_ and PM_10_ levels were 12.9 and 24.3 μg m^−3^. Overall, 29% of personal and indoor PM_2.5_ samples and 33% of personal and indoor PM_10_ samples exceeded WHO 24-h air quality guidelines (15 μg m^−3^ PM_2.5_, 45 μg m^−3^ PM_10_). The factors most strongly associated with personal and indoor PM were household members working in agriculture and regional ambient PM measures.

## 1. Introduction

Exposure to airborne particulate matter (PM) from outdoor and indoor sources is a major contributor to human disease [1]. Exposure to PM, especially PM_2.5_ (≤2.5 μm in aerodynamic diameter) and PM_10_ (≤10 μm in aerodynamic diameter), has been linked to many adverse health effects, including cardiovascular disease (myocardial infarction, stroke, hypertension) [2–4], chronic respiratory disease [1], neurodegenerative disorders [5–7] type 2 diabetes [8], cancer [9] and preterm births [10]. In 2021, over 212.5 million years of life lost prematurely and 19.1 million years lived with disability were attributed to PM pollution [11].

Air pollution exposure assessments are largely based on outdoor air pollution data collected using satellites and fixed-site monitors [12,13]. Outdoor reference-grade monitors are typically sited to capture exposures in urban areas and more densely-populated suburban areas. Few ground-based, reference-grade outdoor air quality monitors capture pollution in rural agricultural communities. Furthermore, on a national scale, higher rates of poverty in rural census tracts are associated with the nearest regulatory PM_2.5_ monitor being further away [14]. Although agricultural communities often lack local reference-grade monitors, residents face unique air exposures due to proximity to livestock emissions; pesticide applications; farm machinery; harvesting, transportation, and processing of agricultural products; and agricultural residue burning [15].

A second limitation of exposure assessments based on outdoor air pollution data is that a person’s exposure to particulate air pollution is not equivalent to the outdoor level measured by the monitor nearest to that person’s home [16,17]. Most people, especially residents of the U.S., spend the majority of their time indoors and transition between multiple “microenvironments” (e.g., home, work, school, community stores or facilities) throughout the day [18–20]. Indoor environments might simultaneously protect occupants against outdoor pollution while also containing additional pollution sources (e.g., emissions from cooking and space heating as well as take-home exposures introduced by household members who have occupational contact with environmental contaminants).

People living in communities that are disproportionately burdened by air pollution from industrial and agricultural activities have often, through their lived experiences, identified their key concerns as well as actions they would like to see taken to remediate pollution [21]. Researchers studying exposures in these communities have a responsibility to engage with residents to ascertain: (a) which questions residents would like answered and (b) how exposure data can be collected, analyzed, and communicated to help residents address their concerns [22–25]. The present study took place in California, where prior community-engaged research has addressed exposure to air pollution in urban communities [22,26–28], as well as documented residents’ environmental concerns and exposure to pesticides in rural communities [29–31].

The present study was nested within a larger study that aimed to characterize exposures to particulate air pollution in California’s San Joaquin Valley and to evaluate new measurement approaches for full-scale community-based sampling [17]. All rural communities that participated in the larger study were identified by the Central California Environmental Justice Network (CCEJN) based on those communities’: (a) proximity to agricultural operations and (b) past collaboration with CCEJN to address residents’ concerns about those operations. During the larger study, residents of one rural community expressed concerns about changes in air quality associated with large-scale processing and transport activities that took place close to their homes during the August–September harvest of a major local crop (unnamed crop to maintain anonymity of the community). In response to these concerns, the study team conducted an additional measurement campaign in this community to characterize PM exposures in September 2024.

The study community had fewer than 2000 residents as of 2023, most of whom identified as Hispanic or Latino. The American Community Survey 5-year estimate for the poverty rate in the community (~18%) was higher than estimates for California (12%) and the U.S. overall (12.5%) [32]. The nearest outdoor PM_2.5_ and PM_10_ monitors operated by the San Joaquin Valley Air Pollution Control District (SJV APCD) were approximately 8 km and 45 km away, respectively.

We investigated how indoor and personal exposures in this community were influenced by a variety of factors, including regional outdoor (i.e., ambient) air pollution levels, seasonal agricultural activities, household cooling methods and cooking activities, as well as occupational exposures among participants. We hypothesized that the September 2024 harvesting season would be associated with higher indoor and personal exposures to PM_2.5_ and PM_10_ mass, given the proximity of community members’ homes to large-scale processing and transport activities that had been observed to produce visible dust.

## 2. Materials and Methods

### 2.1. Participants and Study Visits

We measured PM exposures among 35 unique adult participants from 18 households. Overall, indoor and personal exposures to PM_2.5_ and PM_10_ among residents of this community were measured at three separate timepoints: 4–7 December 2023 (fall), 17–21 May 2024 (spring), and 6–11 September 2024 (summer harvest). These timepoints were selected to capture residents’ exposures during different seasons, assuming that exposures could be influenced by seasonal variations in both: (a) regional air pollution and (b) pollution from local agricultural operations.

Participants were recruited via outreach from local community leaders. Community leaders identified potentially interested participants, called and/or visited them to introduce the study, and forwarded their contact information, with approval, to the study team for follow-up. Participants were eligible if they lived in the study community, were 18+ years of age, were able to provide informed written consent, were English- and/or Spanish-speaking, and were willing to wear personal monitors and host stationary monitors in their homes for 24 h. We aimed to recruit two adults per household so that we could assess PM_2.5_ exposure for one and PM_10_ exposure for the other. If only one participant was recruited from a household, that participant was assessed for only PM_2.5_ exposure or only PM_10_ exposure. Not all individuals and households participated in all three campaigns. If an individual participated in multiple measurement campaigns, that individual’s exposure to the same PM size fraction (PM_2.5_ or PM_10_) was measured during each campaign.

Each household was visited once per campaign for up to three total visits to measure PM exposure for all participants from that household during a 24-h period. At the start of each visit, following the consent process, participants responded to a brief survey and then received air quality monitors (UPAS v2.1 PLUS, Access Sensor Technologies, Fort Collins, CO, USA) that were pre-programmed to sample for 24 h before shutting off automatically. Field staff returned the next day, approximately 24 h later, to ask follow-up survey questions and collect the monitors. Survey questions and response options have been published (supplemental information of Li et al.’s 2025 paper) [17]. At the completion of each study visit, each participant received $50 in cash. An individual could receive a total of up to $150 if that individual participated in all three measurement campaigns. All human subjects research protocols were reviewed and approved by CSU’s Institutional Review Board (protocol #2527).

### 2.2. Personal and Indoor Air Quality Measurements

We measured 24-h-average indoor and personal exposures to PM_2.5_ and PM_10_ by sampling PM onto filters and performing gravimetric analysis on those filter samples. Each PM sample was collected onto a 37-mm PTFE membrane filter (PT37P-PF03, Measurement Technology Laboratories, Minneapolis, MN, USA) using an Ultrasonic Personal Aerosol Sampler (UPAS) v2.1 PLUS (Access Sensor Technologies, Fort Collins, CO, USA). The UPAS is a wearable device that samples PM through a size-selective inlet and onto a filter, using a quiet piezoelectric pump, developed to help reduce cost, noise, and weight while maintaining precision, accuracy, and reliability of PM measurements [19]. Each UPAS v2.1 PLUS was equipped with either a PM_2.5_ or PM_10_ inlet and programmed to sample air at 2 L min^−1^ for 24 h. The extent to which the size-specific collection efficiency of the UPAS PM_2.5_ and PM_10_ inlets matches the United States Environmental Protection Agency (US EPA) definitions of PM_2.5_ and PM_10_ has been documented in previous publications [19,33]. The sample flow control system on each UPAS was calibrated by the manufacturer shortly before the start of each measurement campaign. Data demonstrating that personal and indoor PM_2.5_ and PM_10_ concentrations measured using UPAS v2.1 PLUS agree with those measured using more conventional personal PM sampling equipment have also been published previously [17].

At the start of each 24-h sample, each participant was fitted with a small sling backpack that had the UPAS v2.1 PLUS affixed to the outside of the cross-body strap to measure the participant’s personal exposure to PM_2.5_ or PM_10_. The UPAS inlet faced away from the body and was positioned near the participant’s breathing zone. A small battery pack (V44, Voltaic Systems, Brooklyn, NY, USA) was placed inside each backpack and connected to the UPAS v2.1 PLUS to extend the UPAS runtime with the selected sampling settings to 24 h. Participants were instructed to remove the backpack only when bathing and sleeping but to always keep the backpack nearby. A second UPAS v2.1 PLUS, with the same inlet as the sampler on the backpack (PM_10_ or PM_2.5_) was placed in a central location in the participant’s home to measure the indoor PM level concurrently. This second UPAS was placed in the living room (or another non-bathroom, non-bedroom area) and away from windows or potential PM sources (such as stovetops). At least one field blank filter was collected with a UPAS v2.1 PLUS per day of sampling. Each field blank filter was installed in a UPAS, transported to the field during home visits (without ever turning on the monitor), and then removed from the UPAS at the end of the day.

Each sample and field blank filter was pre- and post-weighed to the nearest 1 μg on a microbalance (XS3DU, Mettler-Toledo, Columbus, OH, USA) to determine the mass of PM_2.5_ or PM_10_ accumulated on the filter [34]. A PM sample mass below the limit of detection (LOD) was replaced with the $LOD/\sqrt{2}$. The LODs for gravimetric analysis of the sample filters were 17, 23, and 29 μg for the December 2023, May 2024, and September 2024 campaigns, respectively. The corresponding LODs of 24-h-average PM concentrations were 6.0, 7.9, and 10 μg m^−3^, respectively, given that all samples were collected at 2 L min^−1^. Full details on laboratory analysis of the filters have been published previously [16].

### 2.3. Other Variables

Survey responses were collected in-person during study visits using Research Electronic Data Capture (REDCap, Colorado Clinical & Translational Sciences Institute, Aurora, CO, USA), a secure, web-based electronic data collection application [35]. Survey questions at the participant-level included basic demographic information, such as participants’ race (Black, American Indian or Alaska Native, Asian, Native Hawaiian or Other Pacific Islander, White, Other), ethnicity (Hispanic/Latino(a) or Non-Hispanic), sex (male, female, non-binary, prefer not to answer), and primary language spoken (English, Spanish). Participants also answered questions regarding whether they were employed in agriculture and/or had occupational dust exposure (yes, no) and/or were occupationally exposed to dust (yes, no).

Household-level surveys were administered to one participant per home. Questions included whether anyone in the home worked in agriculture (yes, no), methods used to cool the home (central air, window units, swamp cooler, open windows, fans, whole house fan or attic fan), time spent cooking with an oven or on a stovetop during the 24-h sample, time spent grilling during the 24-h sample, and vent hood use during cooking [17]. We asked whether any of the following common activities that could contribute to PM pollution took place during the 24-h sample period: burning candles, burning incense, using a scented oil diffuser or burner, using a spray or plug-in air freshener, using an ultrasonic humidifier, sweeping or vacuuming. Given that the Central Valley is often polluted with wildfire smoke during summer months [36], we also asked whether participants had been exposed to wildfire smoke, to their knowledge, in the vicinity of their home during the 24-h sample (yes, no).

The distance from each household to a large nearby agricultural product processing facility was calculated based on two measures: distance to the silos (km) and distance to the centroid of the entire plant (km). The GPS locations of the silos and the entire plant were obtained from OpenStreetMap.

Hourly ambient PM_2.5_ and PM_10_ concentrations measured by beta attenuation monitors at regulatory monitoring sites operated by the SJV APCD were downloaded from the California Air Resources Board website and through the US EPA Air Quality System application programming interface (API) [37,38]. Hourly ambient PM concentrations measured at the nearest regulatory monitoring site for which data were available for ≥75% of the 24-h personal/indoor sample period were time-averaged over the duration of each UPAS v2.1 PLUS filter sample.

Additionally, hourly ambient PM_2.5_ and PM_10_ concentrations measured at regulatory monitoring sites in 2022, 2023, and 2024 were averaged over each day (midnight to midnight local standard time). Subsequently, these 24-h averages were used to calculate the median, 25th percentile, and 75th percentile 24-h average PM_2.5_ and PM_10_ concentrations measured during each month of the year across all regulatory monitoring sites in the San Joaquin Valley air basin.

### 2.4. Data Management and Statistical Analysis

Data for analysis came from four different sources: UPAS v2.1 PLUS filter sample data, the REDCap participant survey data, household distance from the processing plant, and regulatory ambient air monitoring data. All four datasets were joined based on unique household and participant identifying numbers.

We first explored the data on the four PM concentrations (personal and indoor PM_2.5_ and PM_10_) and all covariates descriptively. We assessed Spearman correlations (rho) between 24-h-average personal and indoor PM concentrations. We graphed each PM concentration by sampling campaign in dot and box-and-whisker plots. We reduced the number of covariates of interest by assessing their crude associations with each PM concentration. We assessed diagnostic plots between crude associations to assure the assumptions of linear regression were met and chose to natural log-transform each PM concentration to improve the normality of residuals and homoscedasticity.

To assess up to three repeated measures for each participant and household, we used linear mixed-effects regression models. The outcomes were the personal and indoor 24-h natural log-transformed PM_10_ and PM_2.5_ concentrations, assessed separately. Two key covariates of interest were: (a) ambient concentrations measured at the nearest regulatory monitoring sites and (b) harvest season assessed as a binary variable (harvest season was represented by the September 2024 sampling campaign and non-harvest season was represented by the December 2023 and May 2024 sampling campaigns). All other covariates were also explored as fixed effects to understand their potential associations with each outcome. We included a random effect of participant ID (for personal PM measures) or household ID (for indoor PM measures) to account for non-independence of observations. For covariate selection, we chose variables that showed associations with each outcome in crude regression models, or were well-established risk factors for the outcomes, such as anyone in the household working in agriculture. For final adjusted models, we chose the simplest models that had the fewest variables while still including the two key covariates of ambient PM and harvest season. For all adjusted model beta coefficient results, we calculated percent change in natural log-transformed PM concentration (μg m^−3^) for ease of interpretation: % change = (e^β^ − 1) × 100. All data were managed and analyzed in R (version 2.2.2; R Core Team, Vienna, Austria) within RStudio (version 2024.12.1; Posit, Boston, MA, USA), using the following packages: dplyr [39], ggplot2 [40], and lme4 [41].

### 2.5. Community Engagement and Results Report Back to Participants

We incorporated principles of community-engaged research into this study in several ways: (1) The research questions and study design were established in a manner that prioritized the concerns of the affected community. (2) Community members were actively involved in data collection. (3) Results were discussed with community members and community leaders reviewed and approved this manuscript prior to submission for publication. (4) Authors and community members have working relationships that extend beyond this study. These parties have worked together previously, and will continue to collaborate, to address residents’ environmental concerns.

After each campaign, each household received a customized report of results from the previous campaign outlining PM_2.5_ and PM_10_ exposures measured for participants from the household as well as PM_2.5_ and PM_10_ levels measured inside the home. The reports also included the outdoor PM levels as measured at the nearest regulatory monitoring sites. An anonymized example report is included as Supplementary Material. A member of the study team met with each household after each campaign to discuss the report for that household and answer questions.

## 3. Results

### 3.1. Descriptive Summary

Participant and household characteristics are summarized in Table 1. Participants were mostly female (69%), Hispanic/Latino(a) (100%), and spoke Spanish as their primary language (60%). Agricultural work was common in this community: 36% of personal samples were collected from a participant who worked in agriculture (and/or had occupational dust exposure) and 56% of the 39 unique home sampling visits were conducted at a time when at least one person in the home was working in agriculture (Table 1). The median household distance from the agricultural processing plant silos was 633 m (Q1–Q3: 550–1294). Less than half of homes (39%) were cooled with central air and an air cleaner was used during 33% of home sampling visits. Households tended to use air fresheners and vacuum during the 24-h sample but did not typically burn candles, incense, or scented oils (Table 1).

### 3.2. 24-h Personal and Indoor Air Quality Measurements

Across all three campaigns, we collected 132 PM samples during 39 unique home visits. Six samples (5%) were excluded due to quality assurance issues, including “inlet overloaded” (*n* = 5) and “log file missing” (*n* = 1). A total of 126 PM samples were retained for analysis: personal PM_2.5_ *n* = 31, indoor PM_2.5_ *n* = 31, personal PM_10_ *n* = 30, and indoor PM_10_ *n* = 34. Of these 126 samples, 43, 43, and 40 were collected in December 2023, May 2024, and September 2024, respectively. Of the 126 samples, 9 (7%) had a PM sample mass below the LOD.

Personal exposures to PM_2.5_ and PM_10_ tended to be highest in December 2023, lowest in May 2024, and intermediate during the September 2024 harvest season. Indoor PM_2.5_ and PM_10_ concentrations followed the same trend (Figure 1). Summary statistics for each PM concentration for all sampling campaigns were as follows: personal PM_2.5_ (*n* = 31, median: 11.1 μg m^−3^, Q1–Q3: 8.0–15.4); indoor PM_2.5_ (*n* = 31, median: 12.9 μg m^−3^, Q1–Q3: 8.9–17.0); personal PM_10_ (*n* = 30, median: 45.5 μg m^−3^, Q1–Q3: 31.4–71.2); and indoor PM_10_ (*n* = 34, median: 24.3 μg m^−3^, Q1–Q3: 16.7–36.1). These values are also displayed in Supplemental Materials Table S1. Spearman coefficients showed moderate correlations between personal and indoor PM concentrations across all sampling campaigns, with rho = 0.56 for personal and indoor PM_2.5_ and rho = 0.64 for personal and indoor PM_10_.

Personal and indoor PM_2.5_ and PM_10_ measures are compared to the World Health Organization’s (WHO) 24-h air quality guidelines (15 μg m^−3^ for PM_2.5_ and 45 μg m^−3^ for PM_10_) in Figure 1 [42]. Exposures exceeding these guideline levels may be associated with increased health risks [42]. All measures had observations exceeding the WHO guidelines during each sampling campaign (except indoor PM_10_ in September 2024), despite median personal and indoor PM_2.5_ and indoor PM_10_ concentrations below the guidelines (except one median personal PM_10_ concentration at the guideline in September 2024). Overall, 29% (18/62) of personal and indoor PM_2.5_ samples and 33% (21/64) of personal and indoor PM_10_ samples exceeded the WHO guidelines (Table 2) [42]. The campaign with the highest percentage of samples exceeding the WHO air quality guidelines was December 2023.

Ambient PM_2.5_ concentrations (μg m^−3^) measured at the regulatory monitoring site nearest to the community followed the same seasonal trend as personal and indoor PM_2.5_ samples: highest in December, lowest in May, and intermediate during the September harvest season (Figure S1). Like PM_2.5_, ambient PM_10_ concentrations measured at the nearest regulatory monitoring site were lowest in May. In contrast, ambient PM_10_ concentrations measured at the nearest regulatory monitoring site were highest during the September harvest season (Figure S1). Additionally, all of 24-h ambient PM_10_ concentrations measured during personal and indoor PM_10_ samples, respectively, collected in September exceeded the WHO 45 μg m^−3^ guideline (Figure S1 and Table S3).

When compared to the time-averaged outdoor concentration measured at the nearest regulatory monitoring site over the same 24-h period, personal exposures to PM_2.5_ and PM_10_ tended to be higher in December 2023 (median personal—ambient difference = 8.5 μg m^−3^ for PM_2.5_ and 24.4 μg m^−3^ for PM_10_), more similar but still higher in May 2024 (median personal—ambient difference = 1.1 μg m^−3^ for PM_2.5_ and 0.92 μg m^−3^ for PM_10_), and lower in September 2024 (median personal—ambient difference = −0.88 μg m^−3^ for PM_2.5_ and −6.0 μg m^−3^ for PM_10_). The percentage of personal PM exposures that exceeded the nearest concurrent ambient measurement ranged from 10 to 80%, depending on the size fraction and campaign (Table 2). Indoor PM_10_ concentrations were more likely to be lower than the nearest concurrent ambient measurement.

### 3.3. Adjusted Model Results

Results from unadjusted (crude) linear regression models highlighting which variables were associated with air quality measures can be found in Supplemental Materials (Tables S4 and S5). Based on these results, covariates were included in the adjusted linear regression models to improve precision of results while controlling for potential confounding variables, unique to each air pollutant. Harvest season was always included in the models despite not showing any crude associations with the air pollution measures given the specific interest in this potential exposure variable.

Adjusted model results are shown in Table 3. A key factor associated most strongly with PM measures was anyone in the household working in agriculture, specifically for indoor PM_2.5_ (estimate: 0.55 μg m^−3^, 95% CI: 0.11, 0.98), personal PM_10_ (estimate: 0.49 μg m^−3^; 95% CI: 0.02, 0.95), and indoor PM_10_ (estimate: 0.65 μg m^−3^, 95% CI: 0.28, 1.02). For ease of interpretation, given that exposures were calculated on a natural log scale, indoor PM_2.5_, personal PM_10_, and indoor PM_10_ concentrations were estimated to be 73%, 63%, and 91% higher in households with at least one person working in agriculture compared to households without anyone working in agriculture, respectively (Table 3). Personal PM_2.5_ exposure did not seem to be impacted by anyone in the household working in agriculture (Table 3).

Ambient PM_2.5_ concentrations at the nearest regulatory monitoring site were associated with indoor PM_2.5_ (estimate: 0.09 μg m^−3^, 95% CI: 0.02, 0.16), and suggestively personal PM_2.5_ exposures (estimate: 0.07 μg m^−3^, 95% CI: −0.02, 0.16) (Table 3). This result can be understood as indoor PM_2.5_ concentrations increased by 10% per 1 μg m^−3^ increase in the ambient PM_2.5_ concentration, suggesting outdoor-to-indoor PM_2.5_ infiltration (Table 3). Personal and indoor PM_10_ concentrations were not associated with the ambient PM_10_ concentration measured at the nearest regulatory monitoring site (Table 3). The only other factor that impacted personal PM measures was use of a household air cleaner, which was associated with increased personal PM_2.5_ exposures. People that reported using an air cleaner in their homes had, on average, 86% higher personal PM_2.5_ exposures compared to those who did not use an air cleaner (Table 3). No other factors that we measured were significantly associated with indoor and personal PM_2.5_ and PM_10_ measures.

## 4. Discussion

Agricultural communities face unique environmental exposures yet may be underserved by government air monitoring networks. In our study community, the nearest regulatory monitoring sites were ~8 km away for PM_2.5_ and ~45 km away for PM_10_, which likely made it difficult for monitors at these sites to reliably capture localized pollution from unique sources such as harvest activities, or transportation to and from the processing facility. Personal and indoor monitoring is important to capture exposures from near-home sources; day-to-day activities (such as commuting, cooking, or home cleaning); as well as pollution encountered at work, school, eateries, and other locations [18]. Easy-to-deploy personal monitors, such as the UPAS v2.1 PLUS used in this study, enable personal and household-level data collection at finer spatial and temporal scales [17,19].

The median 24-h-average personal PM_2.5_ exposure measured in the present study for adults living in the rural San Joaquin Valley (11.1 μg m^−3^; Q1–Q3: 8.0–15.4), many of whom worked in agriculture and/or had occupational exposure to dust, was higher than that measured in 2012–2014 for adults living in a mid-sized U.S. city (Fort Collins, CO) who had no occupational exposure to dust or fumes (6.6 μg m^−3^; Q1–Q3: 4.9–10.2) [16]. The higher exposures measured in the present study, combined with the fact that having a household member who worked in agriculture was a significant predictor of higher PM_2.5_ exposure, suggests that occupational exposure (even secondary occupational exposure) is an important contributor to PM_2.5_ exposure in this community. The median 24-h-average personal PM_2.5_ exposure measured in the present study was lower than the medians calculated in a systematic review of personal PM_2.5_ exposures measured in the past 30 years among individuals living in urban (18.6 μg m^−3^; Q1–Q3: 11.4–26.3; 122 groups measured) and rural areas (20.8 μg m^−3^; Q1–Q3: 18.3–39.3; 15 groups measured) of high-income countries across the world [43]. The measurements included in this systematic review from rural areas of high-income countries were all collected between 1996 and 2011, and it is probable that personal exposures to PM_2.5_ in high-income countries have declined generally over the past 10–30 years. For comparison, the national annual-average ambient PM_2.5_ concentration in the U.S. declined by 37% between 2000 and 2023 [44].

Further, our study’s mean personal PM_2.5_ exposure of 15.0 μg m^−3^ (SD: 14.6) and mean indoor PM_2.5_ level of 18.4 μg m^−3^ (SD: 20.3) were consistently twice as high as concentrations measured by ambient monitors throughout the U.S. The 2023 mean seasonally-weighted annual average ambient PM_2.5_ concentration was 8.6 μg m^−3^ for a national sample of 356 U.S. sites and 8.2 μg m^−3^ for 79 sites in the Western region (which includes California) [44]. In 2019, the annual mean PM_2.5_ concentration in rural U.S. census tracts was 6.4 μg m^−3^ (SD: 1.3) [45]. Hispanic or Latino(a) residents of the U.S. are known to be disproportionately burdened by exposure to ambient PM_2.5_ pollution compared to non-Hispanic white residents [46]. Despite this known disparity, the mean exposures we measured were much higher than the annual mean ambient PM_2.5_ concentrations calculated for urban and rural census tracts with large Hispanic/Latino(a) populations in 2019: 8.0 μg m^−3^ (SD: 1.7) and 6.2 μg m^−3^ (SD: 1.6), respectively [43]. Our results support the idea that personal and indoor levels of PM_2.5_ often exceed ambient measures and that fixed-site regulatory monitors might not accurately represent true exposures [18,47]. Measurements at the regulatory monitoring sites closest to the study community tended to underestimate indoor PM_2.5_ concentrations (median personal—ambient difference = 3.6 μg m^−3^), personal PM_2.5_ exposures (median difference = 0.64 μg m^−3^), and personal PM_10_ exposures (median difference = 2.2 μg m^−3^).

Adjusted mixed effects regression models indicated that, in this community where residents lived close to agricultural production and processing activities, having a household member who worked in agriculture and regional ambient PM levels were more strongly associated with indoor and personal PM levels than the harvest season itself. These findings were consistent with our previous study among San Joaquin Valley households within 61 m of agricultural fields, where secondary occupational exposure to agricultural work was a key factor associated with organophosphate pesticides in household dust [31]. This finding has been supported across other studies that suggest agricultural workers may bring home environmental contaminants through a “take-home” route via clothing, shoes, skin, tools, and vehicles, with heightened pesticide exposure among farmworkers and their families during spray seasons [48]. While our exposures of interest in this study were PM_2.5_ and PM_10_ mass, these consistencies highlight the importance of potential occupational-to-residential exposure pathways. Dust and fine particles can adhere to the worker, be brought into the home, and then be resuspended in indoor air through movement, cleaning, or air currents—potentially increasing personal and indoor PM exposures.

Some of our results were unexpected. For example, use of a household air cleaner was associated with increased personal PM_2.5_ exposures. This result was unexpected because air cleaners are designed to reduce indoor PM concentrations and, consequently, personal exposures. It is conceivable that this result was due to reverse confounding, in which individuals who were aware that they were being exposed to higher levels of PM pollution were more motivated to use air cleaners [49–51].

Additionally, contrary to our hypothesis, we found that the September harvest season was not associated with higher indoor PM levels or personal exposures to PM, despite: (a) residents’ and study team members’ observations of outdoor dust associated with processing and transportation of agricultural products during the harvest and (b) higher outdoor PM_10_ levels measured at the nearest regulatory monitoring site during the September 2024 harvest season compared to during the earlier, non-harvest season sampling campaigns (see Figures S1 and S2). It is possible that we observed this result because concerns about processing and transport activities caused residents to take actions to protect themselves from PM exposure.

Lastly, our finding of an association between regional ambient PM_2.5_ levels and indoor PM_2.5_ concentrations (as well as, suggestively, personal PM_2.5_ exposures) has also been supported by other studies. Ambient PM can infiltrate indoor spaces through doors, windows, poorly-sealed structures, or via cooling systems that draw in outdoor air without filtering it thoroughly [22,52]. Overall, personal exposures to PM_2.5_ and PM_10_ were highest in the study community in December, when regional ambient PM_2.5_ pollution in the Central Valley tends to be highest [36]. When presented with these results, community members acknowledged that the higher exposures in December were consistent with their existing knowledge of seasonal variations in regional ambient PM_2.5_ pollution.

### Strengths and Limitations

This study has several strengths. A key strength was the community-engaged approach in which residents’ concerns drove research questions and residents took the first steps in participant recruitment. After each sampling campaign, study team members presented and discussed in-home and personal measurements from the previous campaign, as well as advice for reducing exposure to PM pollution, one-on-one with each household.

Additional strengths of the study included repeated sampling at three timepoints, which captured seasonal variations in exposure. The sampler deployed in this study, the UPAS v2.1 PLUS, allowed us to collect high-resolution, localized data in a region that otherwise has limited air quality monitoring. We used surveys to collect data on numerous participant and household characteristics, which made it possible to assess and adjust for meaningful covariates in the multivariable models. Lastly, our statistical approach using mixed-effects models accounted for participant- and household-level clustering, thus enhancing model validity.

Several limitations of this study are also important to note. The relatively small sample size of 35 participants from 18 households may have limited our statistical power to detect smaller effects from covariates that may have otherwise influenced PM exposures. The small sample size also limited the generalizability of our findings to other study populations that are not similar to the one studied here. The 24-h sampling duration, although repeated three times, was still a relatively small window of exposure that may not have fully captured longer-term exposure patterns or variability in PM levels. While we explored many participant and household characteristics in their associations with PM levels, there were potentially other confounders that we did not measure that could have led to residual confounding.

Finally, a key limitation of this study is that we only measured a limited subset of air quality metrics, namely PM_2.5_ and PM_10_ mass. Our result that the harvest season had no measurable impact on indoor levels of and personal exposures to PM_2.5_ and PM_10_ mass does not mean that there were no adverse environmental exposures among residents of this community stemming from agricultural product processing and transportation activities that took place during the harvest season. The harvest season could be associated with exposures to other pollutants that we did not measure, including pesticides and bioaerosols.

## 5. Conclusions

This study investigated community members’ concerns about their exposure to PM_2.5_ and PM_10_ pollution and possible associations with seasonal activities at a large agricultural processing facility located close to their homes in California’s San Joaquin Valley. We found that the September 2024 harvest season was not associated with indoor or personal exposures to PM_2.5_ or PM_10_ mass. Although this result was contrary to one of our hypotheses, it does not imply that the residents experience no adverse impacts from the nearby processing facility, as we did not quantify an exhaustive list of potential environmental impacts.

The factors most strongly associated with indoor and personal PM exposures were: (1) having anyone in the household who worked in agriculture and (2) regional ambient PM_2.5_ pollution. Having anyone in the household who worked in agriculture was associated with 73%, 63%, and 91% increases in 24-h-average indoor PM_2.5_, indoor PM_10_, and personal PM_10_ exposures, respectively. A 1 μg m^−3^ increase in the 24-h-average ambient PM_2.5_ concentration measured at the nearest outdoor monitoring site was associated with a 10% increase in the 24-h-average indoor PM_2.5_ level. These findings highlight the complex and multifactorial nature of air pollution in agricultural communities, where exposures can arise from both occupational and environmental sources.

The findings presented here also have implications for policy and household-level mitigation efforts. For example, encouraging individuals who work in agriculture to change out of work clothes and shoes before returning home (if they do not do so already) could mitigate secondary occupational exposures. Additionally, given that seasonal trends in the PM_2.5_ and PM_10_ exposures we measured in this study were consistent with seasonal trends in outdoor PM_2.5_ pollution across the Central Valley (i.e., highest in December, much lower in May, and intermediate in September), we would expect efforts to reduce PM exposure in the study community to benefit from: (a) government policies aimed at reducing regional sources of wintertime ambient PM_2.5_ pollution as well as (b) household-level interventions that focus specifically on the cooler months of December and January.

## Supplementary Material

Supplementary Material

Example Participant Report

**Supplementary Materials:** The following supporting information can be downloaded at: https://www.mdpi.com/article/10.3390/environments12100348/s1, Figure S1: Box and dot plots display distributions of 24-h ambient PM_2.5_ and PM_10_ concentrations measured at the nearest regulatory monitoring sites concurrently with personal and indoor samples of the same PM size fraction collected in a rural agricultural community in the San Joaquin Valley, California in December 2023, May 2024, and the September 2024 harvest season. Each point represents the outdoor measurement over the same duration of a 24-h sample from one participant or household. The red dotted horizontal lines indicate the WHO 24-h air quality guidelines: 15 μg m^−3^ for PM_2.5_ and 45 μg m^−3^ for PM_10_.^27^ Boxplots show the median (center line), interquartile range (IQR; box edges = 25th and 75th percentiles), and whiskers extending to 1.5 × IQR; Figure S2. Monthly variability in 24-h average outdoor particulate matter concentrations measured at regulatory monitoring sites operated across the San Joaquin Valley Air Basin. Points and lines indicate the median of all 24-h-averages (midnight to midnight local standard time) measured during that month in 2022–2024. The lower and upper edges of the shaded area represent the 25th and 75th percentiles, respectively; Table S1. Summary statistics for 24-h personal and indoor PM_2.5_ and PM_10_ concentrations (μg m^−3^) measured across study visits (December 2023, May 2024, and September 2024) for 35 participants from 18 households, agricultural community, San Joaquin Valley, California; Table S2. Summary of 24-h ambient PM concentrations (μg m^−3^) measured at the regulatory monitoring sites nearest to the study community concurrently with personal and indoor samples; Table S3. Percentage of 24-h ambient PM concentrations that exceeded the WHO air quality guidelines (AQG) when averaged over personal and indoor sampling times during each campaign; Table S4. Crude mixed-effects regression (with a random effect of participant ID) model associations between harvest season, participant and household characteristics, and natural-log-transformed personal PM exposures (outcome); Table S5. Crude mixed-effects regression (with a random effect of household ID) model associations between harvest season, household characteristics, and natural-log-transformed indoor PM concentrations (outcome).

## Figures and Tables

**Figure 1.**
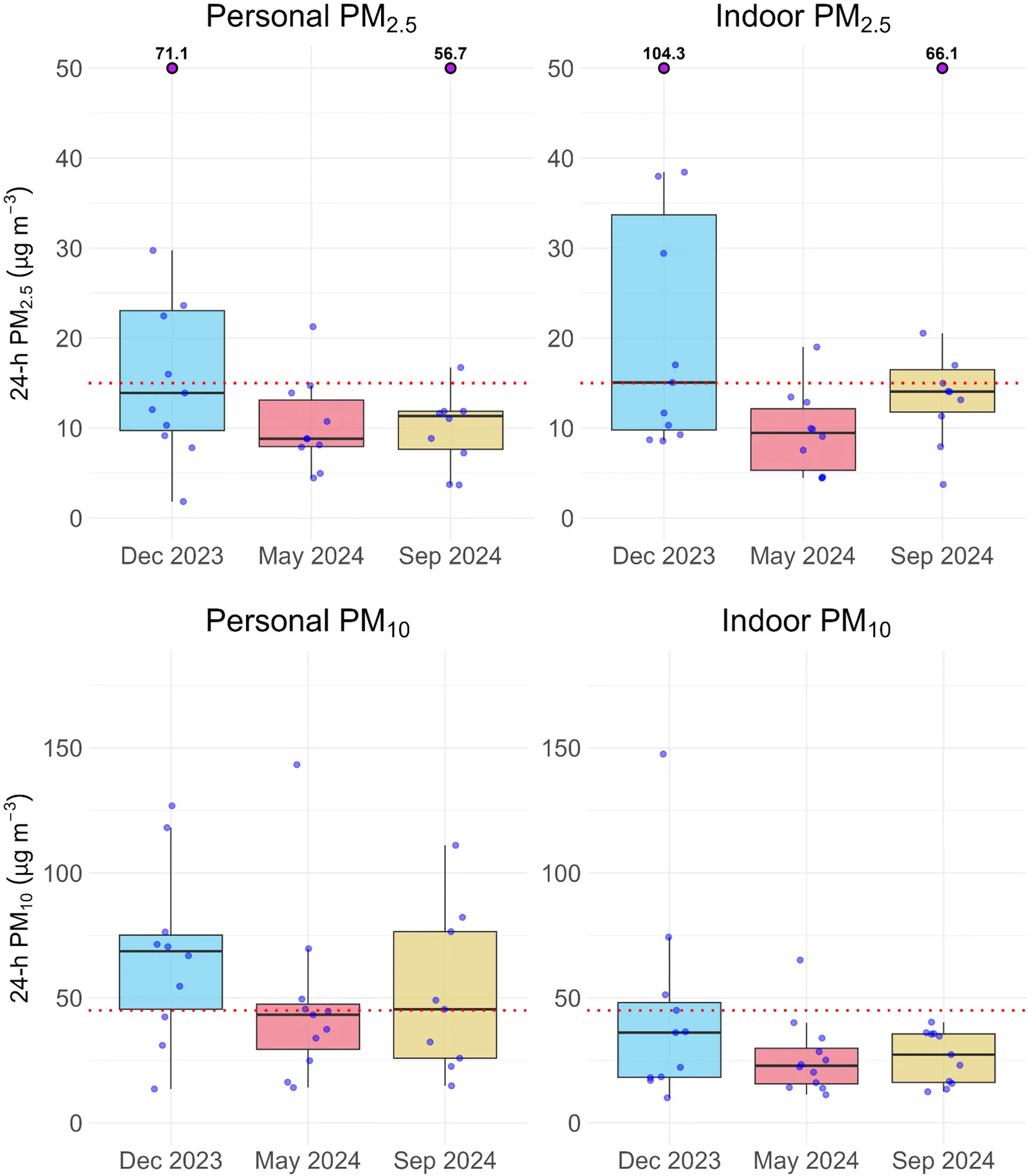
Box and dot plots display distributions of 24-h personal and indoor PM_2.5_ and PM_10_ concentrations measured in a rural agricultural community in the San Joaquin Valley, California in December 2023 (non-harvest season), May 2024 (non-harvest season), and September 2024 (harvest season). Each point represents a 24-h sample from one participant or household. The red dotted horizontal lines indicate the WHO 24-h air quality guidelines: 15 μg m^−3^ for PM_2.5_ and 45 μg m^−3^ for PM_10_. Boxplots show the median (center line), interquartile range (IQR; box edges = 25th and 75th percentiles), and whiskers extending to 1.5 × IQR. Purple data points indicate PM_2.5_ concentrations that exceeded 50 μg m^−3^.

**Table 1.** Descriptive summary of characteristics by participant, household (HH), and sample, December 2023-September 2024 (*n* = 35 unique participants, *n* = 61 personal PM samples, *n* = 18 unique households, *n* = 39 unique home visits, *n* = 126 total PM samples).

Sampling Campaign Dates	*n* = 35 Unique Participants *n* = 18 Unique Households

4–7 December 2023 (non-harvest season)	22 participants, 13 HH
17–21 May 2024 (non-harvest season)	22 participants, 14 HH
6–11 September 2024 (harvest season)	22 participants, 12 HH

**Participant Characteristics**	***n* = 35 unique participants**

Sex: Female, *n* (%)	24 (69%)
Ethnicity: Hispanic/Latino(a), *n* (%)	35 (100%)
Primary language spoken: Spanish, *n* (%)	21 (60%)

**Participant Characteristics**	***n* = 61 personal PM samples**

Works in agriculture and/or occupational dust exposure: Yes, *n* (%)	22 (36%)
Exposed to wildfire smoke in past 24 h: Yes, *n* (%)	3 (5%)

**Household Characteristics**	***n* = 18 unique households**

Household distance from processing facility silos (m): Median (Q1-Q3)	630 (550–1300)
Household cooling with central air: Yes, *n* (%)	7 (39%)

**Household Characteristics**	***n* = 39 unique home visits**

Any household member working in agriculture: Yes, *n* (%)	22 (56%)
Time spent cooking using stovetop or oven during 24-h sample, *n* (%)	
Not at all or less than 30 min	6 (15%)
30 min to 2 h	21 (54%)
More than 2 h	12 (31%)
Vent used that exhausted outside when cooking: Most of the time or always when cooking during 24-h sample, *n* (%)	23 (59%)
Used household air cleaner during 24-h sample: Yes, *n* (%)	13 (33%)
Burned candles, incense, or scented oil in home during 24-h sample: Yes, 1 or more, *n* (%)	6 (15%)
Used a scented oil diffuser, spray air freshener, plug-in air freshener, swept, or vacuumed in home during 24-h sample: Yes, 1 or more, *n* (%)	31 (79%)

**Table 2.** Percentage of 24-h personal and indoor PM_2.5_ and PM_10_ concentrations per campaign that exceeded: (a) WHO 24-h air quality guidelines (15 μg m^−3^ for PM_2.5_ and 45 μg m^−3^ for PM_10_) and (b) 24-h-average outdoor concentrations measured concurrently by the nearest monitor operated by the San Joaquin Valley Air Pollution Control District (SJV APCD).

Measure	December 2023 (Non-Harvest)		May 2024 (Non-Harvest)		September 2024 (Harvest)	
	WHO AQG	SJV APCD	WHO AQG	SJV APCD	WHO AQG	SJV APCD
Personal PM_2.5_	5/11 (45%)	7/11 (64%)	1/10 (10%)	8/10 (80%)	2/10 (20%)	4/10 (40%)
Indoor PM_2.5_	6/11 (55%)	8/11 (73%)	1/10 (10%)	5/10 (50%)	3/10 (30%)	8/10 (80%)
Personal PM_10_	7/10 (70%)	8/10 (80%)	4/11 (36%)	6/11 (55%)	5/9 (56%)	2/9 (22%)
Indoor PM_10_	4/11 (36%)	5/11 (45%)	1/12 (8%)	2/12 (17%)	0/11 (0%)	0/11 (0%)

**Table 3.** Adjusted models. Adjusted associations in mixed effects linear regression between 24-h-average natural-log-transformed PM measures and predictors^1^.

Term	Estimate	CI Lower	CI Upper	% Change^2^	% Change CI Lower	% Change CI Upper

		Personal PM_2.5_ (*n* = 31)				

Ambient PM_2.5_	0.07	−0.02	0.16	7.34	−1.99	17.55
Harvest season (reference is "non-harvest")	−0.02	−0.60	−0.55	−2.30	−45.01	73.61
Anyone in the home working in agriculture (reference is "none")	0.27	−0.23	0.77	30.51	−20.80	115.06
Household use of an air cleaner (reference is "none")	0.62	0.06	1.18	86.43	6.53	226.28

		Indoor PM_2.5_ (*n* = 31)				

Ambient PM_2.5_	0.09	0.02	0.16	9.58	2.41	17.27
Harvest season (reference is "non-harvest")	−0.07	−0.47	0.32	−7.02	−37.37	38.05
Anyone in the home working in agriculture (reference is "none")	0.55	0.11	0.98	72.65	11.52	167.28

		Personal PM_10_ (*n* = 30)				

Ambient PM_10_	0.00	−0.02	0.03	0.39	−2.28	3.13
Harvest season (reference is "non-harvest")	− 0.15	−0.87	0.58	−13.54	−58.11	78.47
Anyone in the home working in agriculture (reference is "none")	0.49	0.02	0.95	62.54	1.69	159.80

		Indoor PM_10_ (*n* = 34)				

Ambient PM_10_	0.001	−0.01	0.02	0.11	−1.28	1.52
Harvest season (reference is "non-harvest")	−0.09	−0.60	0.42	−8.65	−44.94	51.58
Anyone in the home working in agriculture (reference is "none")	0.65	0.28	1.02	91.27	32.13	176.88

1 Adjusted for all other fixed effects listed for the given measure and a random effect for participant or household.

2 Percent change calculation: % change = (e^β^ – 1) × 100.

## Data Availability

The raw data supporting the conclusions of this article will be made available by the authors on request.

## Abbreviations

The following abbreviations are used in this manuscript:

CCEJN: Central California Environmental Justice Network
CI: Confidence interval
CSU: Colorado State University
GSD: Geometric standard deviation
PM_2.5_: fine particulate matter with an aerodynamic diameter of 2.5 μm of less
PM_10_: particulate matter with an aerodynamic diameter of 10 μm or less
SD: Standard deviation
SJV APCD: San Joaquin Valley Air Pollution Control District
UPAS: Ultrasonic Personal Aerosol Sampler
WHO: World Health Organization
