# Supplementary material for: Particulate Matter Pollution in an Agricultural Setting: A Community-Engaged Research Study
Source: Environments (Basel). Author manuscript; Available in PMC 2026 Nov 6. (PMC13739422; doi:10.3390/environments12100348)
Supplement: Supplementary file 1 [file NIHMS2215838-supplement-Example_Participant_Report.pdf]

Ultrasonic Personal Air Sampler (UPAS) Study

Thank you! We appreciate your participation in the Ultrasonic Personal Air Sampler (UPAS) Study. For 24 hours, you carried monitors that measured your personal exposure to particulate matter (PM) air pollution. The Colorado State University team set up monitors that measured particulate matter pollution in your home too. The team also used a survey to learn more about your exposure to pollution.

Particulate matter consists of solids or liquids suspended in the air.[1] These particles include:

- Dust
- Dirt
- Soot
- Smoke
- Drops of liquid

Particulate matter can affect your health. We measured two different sizes of particulate matter:

$PM_{2.5}$

These particles are more dangerous. They can get into the deepest part of your lungs or even into your blood. Common sources of $PM_{2.5}$ include traffic, tobacco smoke, cooking, candles, incense, fireplaces, other fuel-burning heat sources, and wildfire smoke.

PM_{10}

These particles are harmful to your health. They can irritate your eyes, nose, and throat. Common sources of PM_{10} are dust from roads, farms, dry riverbeds, construction sites, and mines.

This report will:

- Show you your 24-hour average personal exposure to $PM_{2.5}$ or PM_{10}
- Show you the 24-hour average PM levels in your home
- Compare your results to those from other anonymous study households
- Compare the 24-hour average PM levels in your home to those measured outside by the nearest San Joaquin Valley Air Pollution Control District monitors
- Illustrate your real-time levels of exposure
- Provide recommendations for reducing indoor air pollution

[1] https://www.epa.gov/pm-pollution/particulate-matter-pm-basics

Overall results

On this page, you can see your time-averaged personal exposures to PM and the time-averaged PM levels in your home over the 24-hour period. Tips for interpreting these results:

- If your personal exposure was higher than the level in your home, you were probably exposed to PM somewhere else, like during your commute, at work, or during an activity you did outdoors.
- If the level in your home was lower than outside, you were probably effective in keeping some outdoor pollution out of your home.
- If the level in your home was higher than outside, there might have been additional sources of PM in your home. Cooking, candles, and incense are examples of common indoor sources of PM.
- The dashed lines are limits set by the Environmental Protection Agency (EPA) for outdoor $PM_{2.5}$ and PM_{10} levels. The 24-hour $PM_{2.5}$ limit is 35 μg/m^3. The 24-hour PM_{10} limit is 150 μg/m^3.

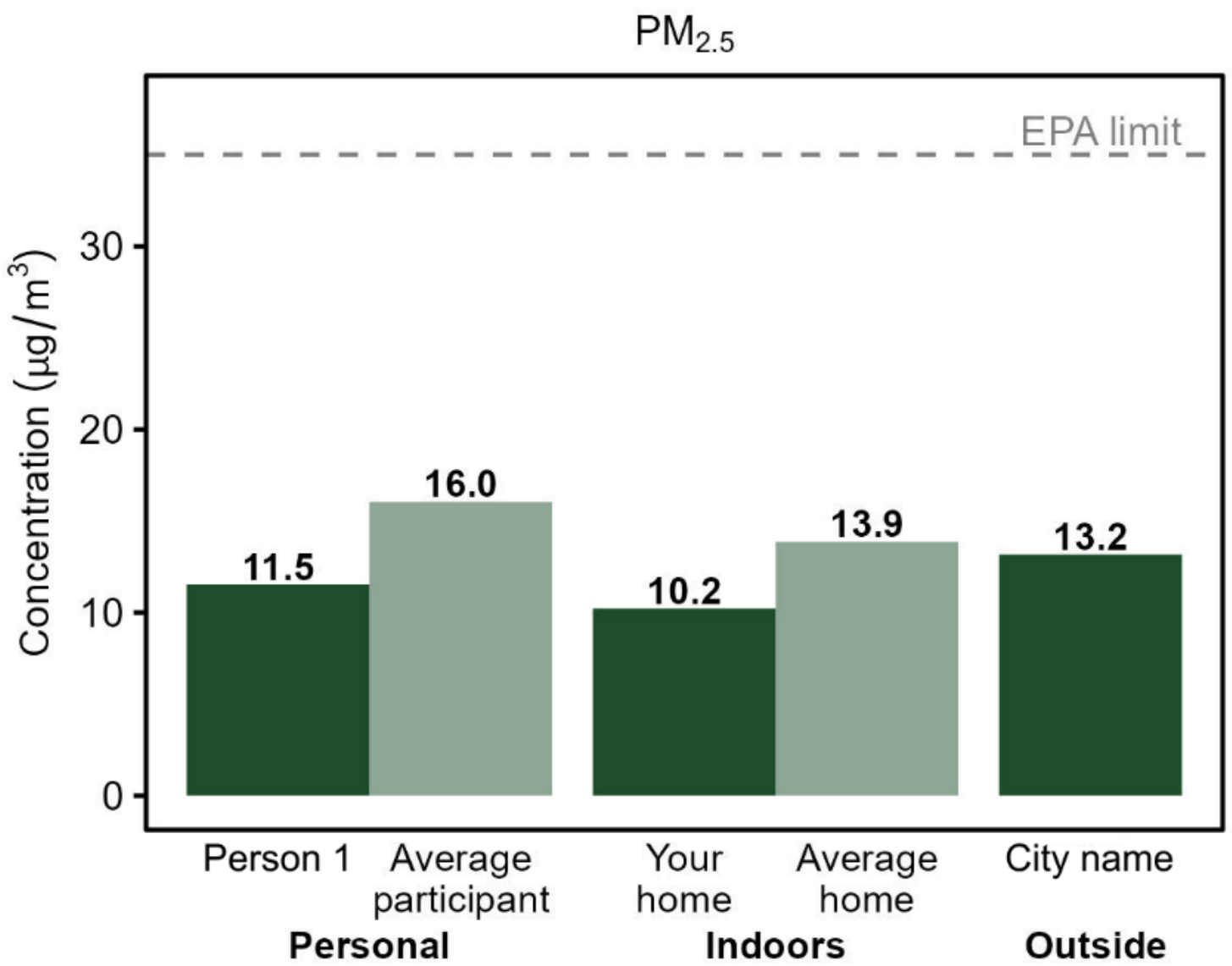

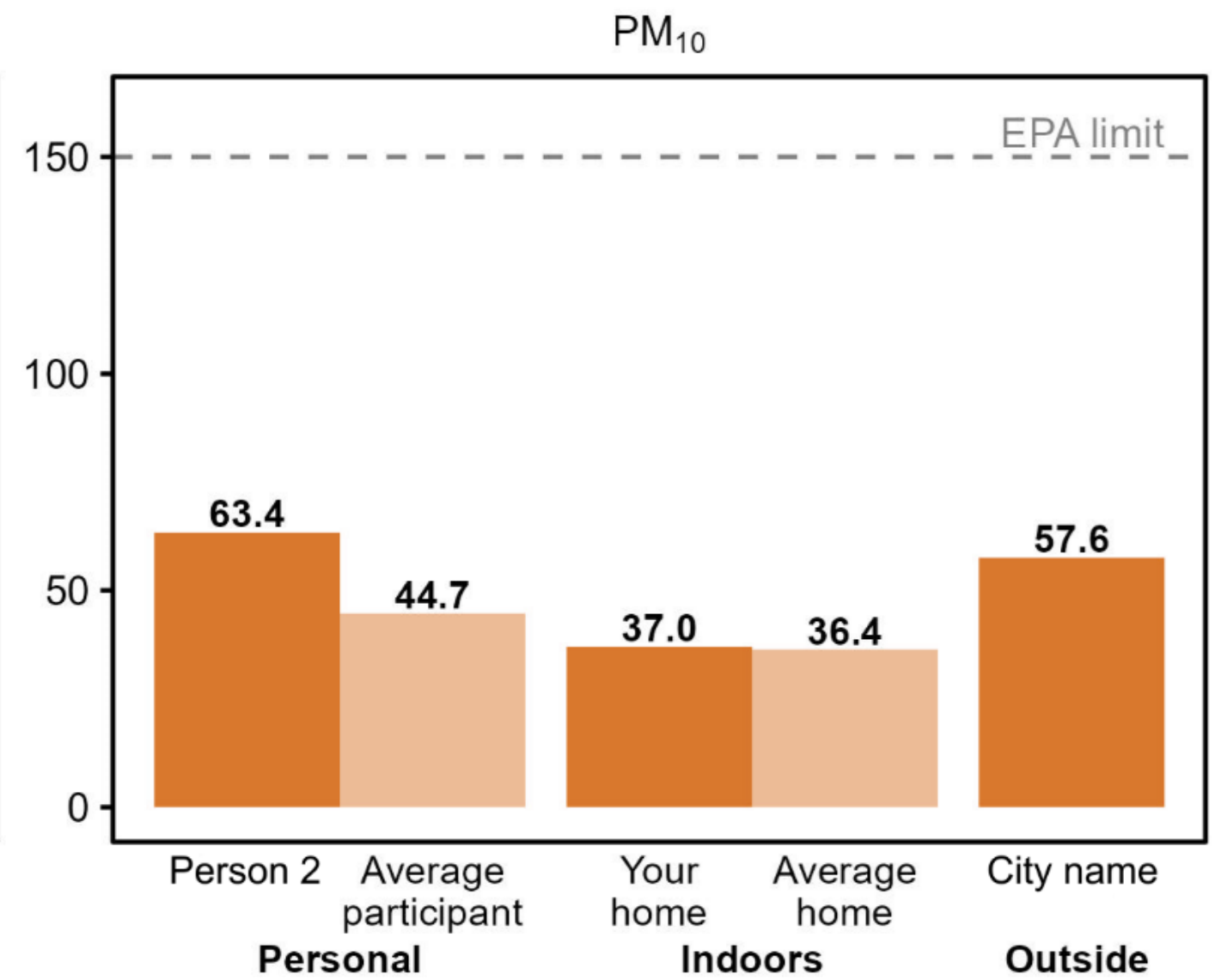

**Personal $PM_{2.5}$ exposure**
Person 1's exposure to $PM_{2.5}$ was…
• **Lower** than the study average and
• **Higher** than the indoor level.
**Indoor $PM_{2.5}$ level**
The $PM_{2.5}$ level in your home was…
• **Lower** than the study average and
• **Lower** than the outdoor level.

**Personal PM_{10} exposure**
Person 2's exposure to PM_{10} was…
• **Higher** than the study average and
• **Higher** than the indoor level.
**Indoor PM_{10} level**
The PM_{10} level in your home was…
• **Similar** to the study average and
• **Lower** than the outdoor level.

Time-resolved results

Below, you can see how the $PM_{2.5}$ level inside your home and your personal exposure changed over the 24-hour period. In this graph, white areas with a sun illustrate daytime. Gray areas with a moon illustrate nighttime. Each vertical dotted line is an hour of the day (for example, 1 PM, 2 PM, 3 PM).

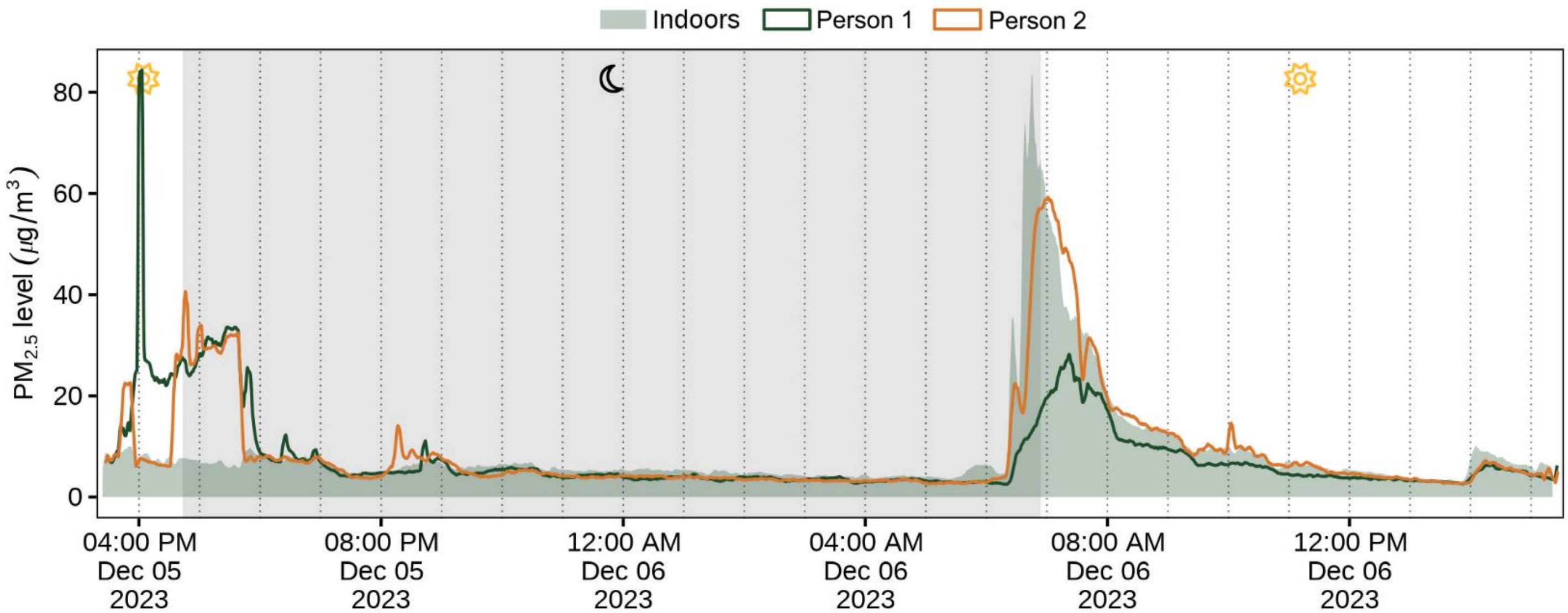

Tips for interpreting these results:

- During times when your personal exposure was similar to the level in your home and had the same trend over time, you were exposed to indoor air pollution in your home.
- Think about what activities might have taken place in and around your home at times when the UPAS measured higher indoor $PM_{2.5}$ levels.
- If there were times when your personal exposure was higher than the level in your home and the trend of your personal exposure did not match the trend in your home, you might have been exposed to particulate matter somewhere else, like during your commute, at work, or during an activity you did outdoors.
- Think about where you were and what you were doing when the UPAS detected higher personal exposure to $PM_{2.5}$.

Recommendations for reducing indoor air pollution

Although particulate matter air pollution can be commonplace within the home, there are measures you can take to reduce indoor air pollution.

Source Control

- A vent above your stove that exhausts outdoors can remove particulate matter from cooking. Always turn on the vent while cooking with your stove.
- Dont burn candles, incense, waxes, or oils in your home.

Ventilation

- Open windows and doors.
- Operate window or attic fans.
- Run a window air conditioner with the vent control open.
- *Take outdoor air quality into consideration before opening windows.*

Cleaning the Air

- If possible, use an air cleaner with a HEPA filter. When selecting a specific model of air cleaner, consider how the size of the room the air cleaner is recommended for compares to the size of the space where you plan to use it.
- You can also build your own DIY air cleaner. Scan the QR code for instructions!
- If you have forced-air heating and/or cooling, install a high-quality filter in the system (for example, a filter with a MERV-13 rating). Replace the filter regularly. If the air quality outside is poor, running your forced air system, even if it's in "fan only" mode, can help clean the air in your home by circulating it through the filter.

Visit AirNow to learn more about the Air Quality Index for outdoor particulate matter and ozone pollution:

https://www.airnow.gov/aqi/aqi-basics/

Questions?

If you have any questions about this document, you can contact the principal investigator, Sheryl Magzamen. Call **970-491-5469** or email **Sheryl.Magzamen@colostate.edu**
