# Supplementary material for: Particulate Matter Pollution in an Agricultural Setting: A Community-Engaged Research Study
Source: Environments (Basel). Author manuscript; Available in PMC 2026 Nov 6. (PMC13739422; doi:10.3390/environments12100348)
Supplement: Supplementary file 2 [file NIHMS2215838-supplement-Supplementary_Material.pdf]

SUPPLEMENTAL MATERIALS

Particulate matter pollution in an agricultural setting: A community-engaged research study

Bonnie N. Young[1], Jessica Tryner[2], Luis Hernandez Ramirez[1], Sherry WeMott[1], Grant Erlandson[1], Xiaoying Li[3], Grace Kuiper[1], Daniel Alan Dean[1], Nayamin Martinez[4], Mollie Phillips[2], John Volckens[3], Sheryl Magzamen[1]*

[1] Department of Environmental and Radiological Health Sciences, Colorado State University, Fort Collins, Colorado 80523, United States
[2] Access Sensor Technologies, Fort Collins, Colorado 80524, United States
[3] Department of Mechanical Engineering, Colorado State University, Fort Collins, Colorado 80523, United States
[4] Central California Environmental Justice Network, Fresno, California 93727, United States

*Corresponding author: Sheryl Magzamen, sheryl.magzamen@colostate.edu

List of items in Supplemental Materials:

Tables

- Table S1. Summary statistics for 24-h personal and indoor $PM_{2.5}$ and PM_{10} concentrations (µg m^{-3}) measured across study visits (December 2023, May 2024, and September 2024) for 35 participants from 18 households, agricultural community, San Joaquin Valley, California.
- Table S2. Summary of 24-h ambient PM concentrations (µg m^{-3}) measured at the regulatory monitoring sites nearest to the study community concurrently with personal and indoor samples.
- Table S3. Percentage of 24-h ambient PM concentrations that exceeded the WHO air quality guidelines (AQG) when averaged over personal and indoor sampling times during each campaign.
- Table S4. Crude mixed-effects regression (with a random effect of participant ID) model associations between harvest season, participant and household characteristics, and natural-log-transformed **personal PM exposures** (outcome)
- Tables S5. Crude mixed-effects regression (with a random effect of household ID) model associations between harvest season, household characteristics, and natural-log-transformed **indoor PM concentrations** (outcome)

Figures

- Figure S1. Box and dot plots display distributions of 24-h ambient $PM_{2.5}$ and PM_{10} concentrations measured at the nearest regulatory monitoring sites concurrently with personal and indoor samples of the same PM size fraction collected in a rural agricultural community in the San Joaquin Valley, California in December

2023, May 2024, and the September 2024 harvest season. Each point represents the outdoor measurement over the same duration of a 24-h sample from one participant or household. The red dotted horizontal lines indicate the WHO 24-h air quality guidelines: 15 μg m^{-3} for $PM_{2.5}$ and 45 μg m^{-3} for PM_{10}.[27] Boxplots show the median (center line), interquartile range (IQR; box edges = 25th and 75th percentiles), and whiskers extending to 1.5×IQR.

- Figure S2. Monthly variability in 24-h average outdoor particulate matter concentrations measured at regulatory monitoring sites operated across the San Joaquin Valley Air Basin. Points and lines indicate the median of all 24-h-averages (midnight to midnight local standard time) measured during that month in 2022–2024. The lower and upper edges of the shaded area represent the 25th and 75th percentiles, respectively.

Table S1. Summary statistics for 24-h personal and indoor $PM_{2.5}$ and PM_{10} concentrations (µg m^{-3}) measured across study visits (December 2023, May 2024, and September 2024) for 35 participants from 18 households, agricultural community, San Joaquin Valley, California.

PM measures	n	Mean	SD	Geometric mean	GSD	Min	Q1	Median	Q3	Max
Personal UPAS $PM_{2.5}$ µg m^{-3}	31	15.00	14.57	11.18	2.12	1.84	8.01	11.06	15.36	71.08
Indoor UPAS $PM_{2.5}$ µg m^{-3}	31	18.35	20.33	13.23	2.11	3.73	8.89	12.87	17.00	104.28
Personal UPAS PM_{10} µg m^{-3}	30	55.15	34.45	45.42	1.92	13.56	31.37	45.47	71.19	143.33
Indoor UPAS PM_{10} µg m^{-3}	34	31.82	25.35	26.26	1.80	10.04	16.72	24.27	36.11	147.53

N: number of observations; GSD: geometric standard deviation; SD: standard deviation: Q1: 25th percentile; Q3: 75th percentile

Table S2. Summary of 24-h ambient PM concentrations (μg m^{-3}) measured at the regulatory monitoring sites nearest to the study community concurrently with personal and indoor samples.

Size fraction (full dataset)	n	Median	Q1-Q3	Min-Max
$PM_{2.5}$ (measured 8 km away)	62	8.4	7.3-12.5	4.9-14.0
PM_{10} (measured 45 km away)	64	41.3	31.2-51.0	28.9-89.4

Q1: 25th percentile; Q3: 75th percentile

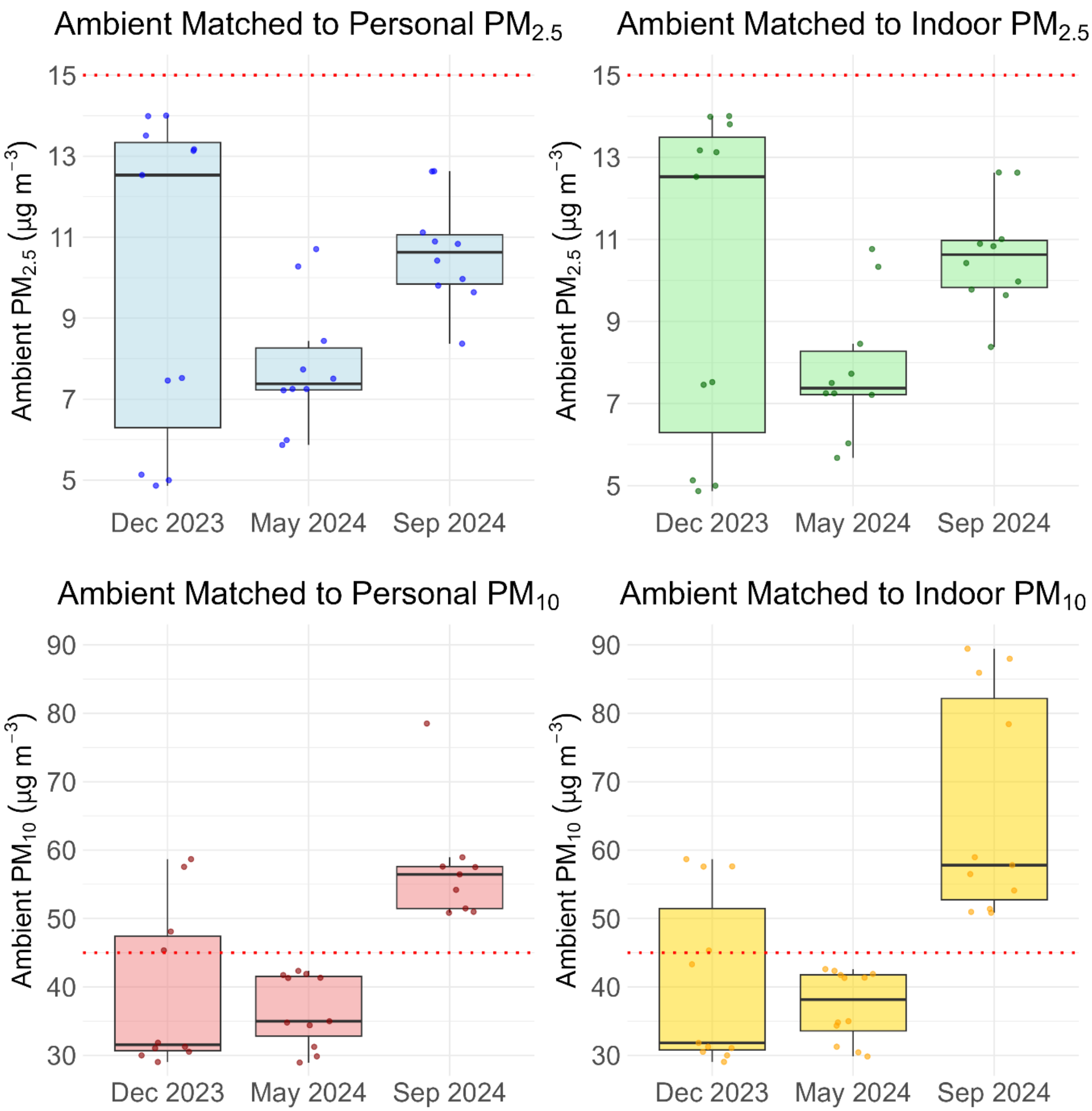

Figure S1. Box and dot plots display distributions of 24-h ambient $PM_{2.5}$ and PM_{10} concentrations measured at the nearest regulatory monitoring sites concurrently with personal and indoor samples of the same PM size fraction collected in a rural agricultural community in the San Joaquin Valley, California in December 2023, May 2024, and the September 2024 harvest season. Each point represents the outdoor measurement over the same duration of a 24-h sample from one participant or household. The red dotted horizontal lines indicate the WHO 24-h air quality guidelines: 15 µg m^{-3} for $PM_{2.5}$ and 45 µg m^{-3} for PM_{10}.[27] Boxplots show the median (center line), interquartile range (IQR; box edges = 25th and 75th percentiles), and whiskers extending to 1.5×IQR.

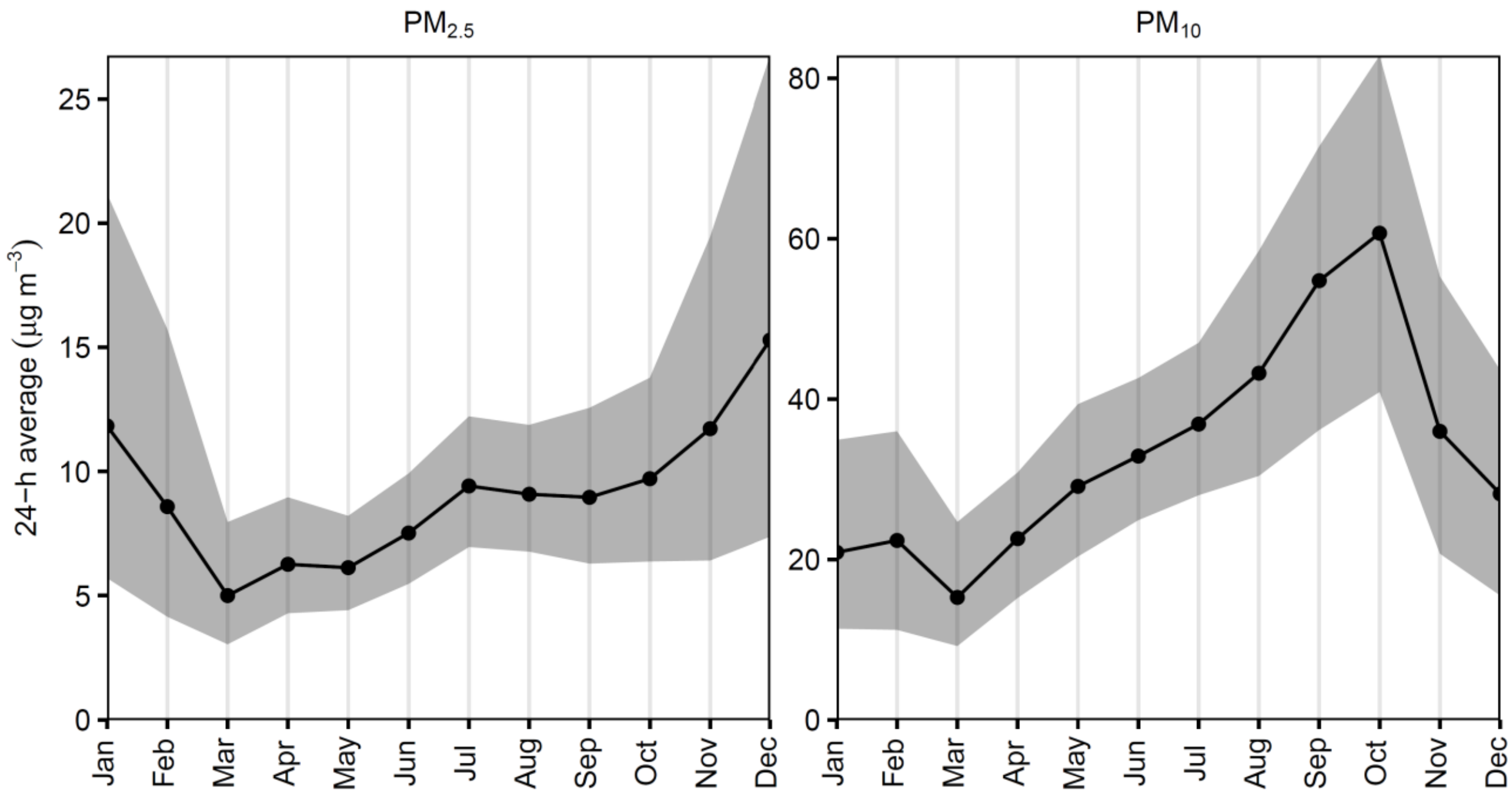

Figure S2. Monthly variability in 24-h average outdoor particulate matter concentrations measured at regulatory monitoring sites operated across the San Joaquin Valley Air Basin. Points and lines indicate the median of all 24-h-averages (midnight to midnight local standard time) measured during that month in 2022–2024. The lower and upper edges of the shaded area represent the 25th and 75th percentiles, respectively.

Table S3. Percentage of 24-h ambient PM concentrations that exceeded the WHO air quality guidelines (AQG) when averaged over personal and indoor sampling times during each campaign

PM Measure	WHO 24-h AQG	Dec 2023 (non-harvest)	May 2024 (non-harvest)	Sep 2024 (harvest)
Ambient $PM_{2.5}$ linked to personal $PM_{2.5}$	15 ug m^{-3}	0/11 (0%)	0/10 (0%)	0/10 (0%)
Ambient $PM_{2.5}$ linked to indoor $PM_{2.5}$	15 ug m^{-3}	0/11 (0%)	0/10 (0%)	0/10 (0%)
Ambient PM_{10} linked to personal PM_{10}	45 ug m^{-3}	4/10 (40%)	0/11 (0%)	9/9 (100%)
Ambient PM_{10} linked to indoor PM_{10}	45 ug m^{-3}	4/11 (36%)	0/12 (0%)	11/11 (100%)

Table S4. Crude mixed-effects regression (with a random effect of participant ID) model associations between harvest season, participant and household characteristics, and natural-log-transformed **personal PM exposures** (outcome).

Term	$PM_{2.5}$			PM_{10}		
	Estimate	CI Lower	CI Upper	Estimate	CI Lower	CI Upper
Ambient PM concentration in the same size fraction (µg m^{-3})	0.07	-0.03	0.16	0	-0.02	0.02
Harvest season (reference is "non-harvest")	-0.1	-0.68	0.47	-0.09	-0.61	0.42
Sex: Male (reference is female)	-0.26	-0.79	0.27	0.37	-0.27	1.02
Primary spoken language: Spanish (reference is English)	-0.14	-0.68	0.4	0.23	-0.28	0.74
Works in agriculture (reference is no)	0.28	-0.27	0.83	**0.52**	**0.06**	**0.98**
Anyone in the house working in agriculture (reference is "none")	**0.34**	**-0.19**	**0.87**	**0.49**	**0.03**	**0.94**
Household use of an air cleaner (reference is "none")	**0.68**	**0.16**	**1.2**	0.17	-0.35	0.69
Household use of central air for cooling (reference is no)	0.24	-0.34	0.81	-0.44	-0.89	0
Combined any PM-generating activities in house in past 24 hours[1] (reference is none)	-0.28	-0.92	0.35	0.32	-0.36	1
Burn-only PM-generating activities in house in past 24 hours[2] (reference is none)	0.5	-0.2	1.21	0.39	-0.25	1.02
Non-burning PM-generating activities in house in past 24 hours[3] (references is none)	-0.42	-1.02	0.17	0.32	-0.36	1
Household distance from agricultural processing facility silos (m)	0	0	0	0	0	0
Vent use of stove hood when cooking that exhausts outside and is used most of the time or always when cooking (reference is no vent or vent doesn't exhaust outside, or never or only sometimes use vent when cooking)	0.09	-0.46	0.64	-0.23	-0.7	0.25

Table S5. Crude mixed-effects regression (with a random effect of household ID) model associations between harvest season, household characteristics, and natural-log-transformed **indoor PM concentrations** (outcome).

	$PM_{2.5}$			PM_{10}		
**Term**	**Estimate**	**CI Lower**	**CI Upper**	**Estimate**	**CI Lower**	**CI Upper**
Ambient PM concentration in the same size fraction (μg m^{-3})	**0.08**	**0.01**	**0.15**	0	-0.01	0.01
Harvest season (reference is “non-harvest”)	0.11	-0.31	0.53	-0.09	-0.43	0.25
Anyone in the house working in agriculture (reference is “none”)	**0.51**	**0.03**	**0.99**	**0.65**	**0.28**	**1.01**
Household use of an air cleaner (reference is “none”)	0.36	-0.09	0.82	0.14	-0.23	0.51
Household use of central air for cooling (reference is no)	0.22	-0.39	0.82	0.09	-0.38	0.56
Combined any PM-generating activities in house in past 24 hours[1] (reference is none)	-0.05	-0.56	0.46	0.22	-0.24	0.68
Burn-only PM-generating activities in house in past 24 hours[2] (reference is none)	0.29	-0.35	0.93	0.44	-0.09	0.97
Non-burning PM-generating activities in house in past 24 hours[3] (references is none)	-0.23	-0.76	0.29	0.22	-0.24	0.68
Household distance from agricultural processing facility silos (m)	0	0	0	0	0	0
Vent use of stove hood when cooking that exhausts outside and is used most of the time or always when cooking (reference is no vent or vent doesn’t exhaust outside, or never or only sometimes use vent when cooking)	0.01	-0.48	0.51	-0.02	-0.43	0.4
